# *De novo* reconstitution of chromatin using wheat germ cell‐free protein synthesis

**DOI:** 10.1002/2211-5463.13178

**Published:** 2021-05-16

**Authors:** Yaeta Endo, Nobuaki Takemori, Szilvia K. Nagy, Kei‐ichi Okimune, Rohinton Kamakaka, Hitoshi Onouchi, Taichi E. Takasuka

**Affiliations:** ^1^ Proteo‐Science Center Ehime University Matsuyama Japan; ^2^ Division of Proteomics Research Proteo‐Science Center Ehime University Toon Japan; ^3^ Department of Molecular Biology Institute of Biochemistry and Molecular Biology Semmelweis University Budapest Hungary; ^4^ Research Faculty of Agriculture Hokkaido University Sapporo Japan; ^5^ Department of Molecular Cell and Developmental Biology University of California at Santa Cruz CA USA; ^6^ Global Institute for Collaborative Research and Education Hokkaido University Sapporo Japan

**Keywords:** ATP‐dependent nucleosome assembly, histone modifications, Mg^2+^ chromatin isolation, post‐translational chromatin assembly, unmodified and soluble histone products, wheat germ cell‐free protein synthesis

## Abstract

DNA is packaged with histones to form chromatin that impinges on all nuclear processes, including transcription, replication and repair, in the eukaryotic nucleus. A complete understanding of these molecular processes requires analysis of chromatin context *in vitro*. Here, *Drosophila* four core histones were produced in a native and unmodified form using wheat germ cell‐free protein synthesis. In the assembly reaction, four unpurified core histones and three chromatin assembly factors (dNAP‐1, dAcf1 and dISWI) were incubated with template DNA. We then assessed stoichiometry with the histones, nucleosome arrays, supercoiling and the ability of the chromatin to serve as a substrate for histone‐modifying enzymes. Overall, our method provides a new avenue to produce chromatin that can be useful in a wide range of chromatin research.

## Graphical abstract

We provide a new method to reconstitute chromatin using wheat germ cell‐free protein synthesis. The *Drosophila* four core histones were produced in a native and unmodified form. In the assembly reaction, four unpurified core histones and three assembly factors (dNAP‐1, dAcf1 and dISWI) were incubated with DNA, followed by Mg^2+^ isolation. The chromatin was evaluated and then used as substrates for histone‐modifying enzymes.

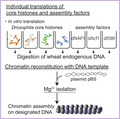

**Abbreviations**
Acf1: ATP‐dependent chromatin assembly factor large subunit
AMP‐PCP: β,γ‐Methylene adenosine 5'‐triphosphate
ISWI: chromatin‐remodeling complex ATPase chain Iswi
MNase: micrococcal nuclease
MS: mass spectrometry
NAP‐1: nucleosome assembly protein NAP‐1
SC‐pBS: supercoiled pBluescript
SRM: selected reaction monitoring

In eukaryotic cells, chromatin structure plays various roles in gene regulation, DNA repair, DNA replication and inheritance of epigenetic information from generation to generation. The basic building block of chromatin is the nucleosome, which contains about 147 bp of DNA wrapped 1.7 times around two copies each of the histones H2A, H2B, H3 and H4 [1, 2]. The basic structure of chromatin is identical in almost all eukaryotes, but histone variants and chemical modifications of the histones introduce a considerable heterogeneity in chromatin structure [3, 4, 5, 6]. Although a tremendous amount of information has accumulated in the past decades regarding the identity of the posttranslational modifications of histones [2], it is extremely challenging to understand their effects on chromatin structure, dynamics and physiological functions. To fully understand the biochemical role of histone modifications on chromatin, it is necessary to reconstitute chromatin *in vitro* with defined components as much as possible.

The reconstitution of chromatin *in vitro* first involves the preparation of the histones in the correct stoichiometry before chromatin assembly. Some of the earliest methods of histone purification involved acid or high‐salt extraction of the intact histones from eukaryotic nuclei followed by reassembly of the octamer and purification before chromatin reconstitution [7, 8, 9]. However, the earlier‐mentioned extraction conditions might denature the proteins and require substantial manipulation. Alternatively, histones can be prepared by the synthesis and purification of recombinant proteins from *Escherichia coli* [10, 11, 12]. In this method, the expressed recombinant histones are needed to be extracted from the inclusion bodies and then refolded.

There are two conventional methods for the reconstitution of chromatin on DNA [13, 14]. In the first series of methods, purified histones from living tissues or recombinant histones are mixed together in equal stoichiometry to form octamers, then mixed with DNA at high‐salt concentrations, followed by step‐wise dialysis resulting in the assembly of nucleosomes [15, 16]. This method is usually unable to evenly space nucleosomes unless the DNA template contains arrays of nucleosome positioning sequences and is primarily used to reconstitute chromatin onto short fragments of DNA [17, 18, 19]. In the second series of methods, chromatin is assembled using chromatin assembly factors, such as histone chaperones and ATP‐dependent chromatin remodeling factors [7, 20, 21, 22]. The methods also require purified histones at equal stoichiometry.

To date, *in vitro*‐reconstituted chromatin has been widely used to investigate fundamental processes of molecular biology, such as transcription [23], gene silencing [24], centromeric chromatin and others [25, 26, 27]. However, purification of histones and chromatin assembly factors from living tissues is time consuming and also suffers additional technical limitations. For example, core histones purified from eukaryotic cells are highly heterogeneous, containing different subtypes, and possess multiple posttranslational modifications, all of which are not desirable for the controlled chromatin reconstitution *in vitro* [8, 28]. In contrast, although recombinant core histones produced in bacterial cells are chemically homogeneous, refolding is necessary after the expression of proteins. For these reasons, recombinant histones are less efficient at forming nucleosomes compared with native histones [22, 29]. Moreover, supplementation of salts and macromolecules, such as polyethylene glycol or bovine serum albumin, during assembly introduces extraneous molecules that could interfere with the downstream processes.

Recently, we developed a novel reconstitution method using a wheat germ cell‐free coexpression system and showed both human and *Drosophila* chromatins were assembled onto a circular DNA in the presence of mRNAs coding four core histones and appropriate chromatin assembly factors [30]. Both supercoiling assay and micrococcal nuclease (MNase) digestion were used to assess the reconstituted chromatins. In this study, we detailed more insights into our reconstitution system by using *Drosophila* chromatin components with regard to chemical modification profiles of synthesized histones, the kinetics of histone production in the cell‐free system and the determination of essential components in the assembly.  Furthermore, we showed that the reconstituted chromatin can be easily isolated via the aid of divalent cations with moderate purity, and the isolated chromatin retained the expected stoichiometry of core histones after recovery. We examined histone modifications on the reconstituted chromatin and found that the chromatin was modified with dGCN5 and dSet8 that resulted in acetylation of nucleosomal H3 and methylation of nucleosomal H4, respectively. The described method might be applicable toward the straightforward preparation of defined chromatin and applicable to a wide range of areas in chromatin structure and function research.

## Materials and methods

### Cell‐free protein synthesis

The methods for cloning of genes from *Drosophila melanogaster* into the pEU expression plasmid vector, preparation of a cell‐free extract from wheat embryos and the transcription and translation reactions via a membrane dialysis device (Slide‐A‐Lyzer, Product #8841; Thermo Scientific, Rockford, IL, USA) were described previously [30, 31, 32]. *In vitro* transcription was performed as previously described using the pEU vector carrying an individual gene [33]. The transcribed reaction mixture was treated with RNase‐free DNase1 (1 U·100 µL^−1^; Nippon Gene, Tokyo, Japan) for 30 min at 37 ˚C and deprotonated by phenol‐chloroform extraction followed by EtOH precipitation. To eliminate residual nucleotides from the transcription reaction, we extensively washed RNA precipitate with 75% EtOH, and mRNA was dissolved in RNase‐free Milli‐Q water. In the dialysis cup mode translation, a 100‐µL translation mixture contained 4 µg of creatine kinase (Roche Diagnostics, GmbH, Mannheim, Germany) and 17 µL of the stock wheat germ extract (*A*_260_ = 240; thus, final 40 *A*_260_ units·mL^−1^) (WEPRO7240H, Lot: 15AQ02; Cell‐free Sciences, Yokohama, Japan). A total of 25 µg of the total amount of mRNA, which was predetermined to be optimum, was used for the individual, or each of 12.5 µg H2A/H2B or H3/H4 and each of 6.25 µg of H2A/H2B/H3/H4 for the cotranslation. The translation reaction was performed by dipping the dialysis device in 5 mL of substrate solution at 15 ˚C for 50 h. The translation mixtures and substrate buffer solution were composed of the following: 30 mm HEPES–KOH (pH 7.6), 100 mm potassium acetate, 2.7 mm magnesium acetate, 0.4 mm spermidine, 2.5 mm DTT, 0.3 mm each amino acid, 1.2 mm ATP, 0.25 mm GTP and 16 mm creatine phosphate. For radiolabeling of the proteins, the translation reaction was performed in the presence of 0.18 MBq·mL^−1^ ^14^C‐leucine (11.1 GBq·mmol^−1^; PerkinElmer, Foster, CA, USA) in the translation mixture and substrate buffer. Concentrations of synthesized histones were determined and adjusted to 4.0 pmol·µL^−1^ for individually expressed ones and each 2.0 pmol·µL^−1^ for coexpressed histones H2A and H2B and also H3 and H4 by diluting with a separately incubated translation mixture in the absence of mRNA. Aliquots of product solutions from the cup device were rapidly frozen in liquid nitrogen and stored at −80 ˚C until use. Solubility (%) of products was the ratio of hot trichloroacetic acid (TCA)‐insoluble radioactivity in the supernatants to that of the total after centrifugation at 10 000 ***g*** for 10 min, measured by liquid scintillator (Ultima Gold XR; Packard). The counting efficiency of ^14^C in proteins on filters was corrected and normalized by using purified ^14^C‐labeled red fluorescent protein in solution. The amount of synthesized protein was calculated by moles of leucine incorporated and the number of residues in each protein. Relative intensities among coomassie brilliant blue (CBB)‐stained or the bands on the autoradiogram were measured by densitometry (Typhoon FLA 7000BGR; GE Health Care, Pittsburgh, PA, USA).

### Wheat germ cell‐free *in vitro* chromatin assembly

Three types of the 30 µL of standard chromatin assembly mixture were prepared by simply mixing translated solutions containing 20 pmol each of individually synthesized four core histones, cotranslated histones, H2A/H2B and H3/H4, or cotranslated all four histones (H2A/H2B/H3/H4) and 2.0 pmol each of the supernatant fraction of dNAP‐1, dISWI and dAcf1 in the standard reaction. The concentration of wheat germ extract in the assembly mixture was adjusted to the final 40 *A*_260_ by adding a separately incubated translation mixture without mRNA, and 5 µg of creatine kinase was supplemented. Unless otherwise stated in the figure legends, an assembly mixture was preincubated with 60 U MNase (Nippon Gene) with 1 mm Ca^2+^ for 30 min at 26 ˚C to digest the endogenous wheat DNA; then the reaction was halted by adding 5 mm EGTA. The assembly reaction was initiated by adding 1 µg supercoiled pBluescript (SC‐pBS) (pBluescript^SK+^; Agilent, Santa Clara, CA, USA) and incubated for 3 h at 26 ˚C. A β,γ‐methyleneadenosine 5′‐triphosphate (AMP‐PCP) was used as nonhydrolyzable ATP analogue (Wako, Osaka, Japan). Topoisomerase I and II inhibitors, camptothecin (Wako) and ICRF‐193 (Sigma‐Aldrich, St. Louis, MO, USA), were purchased.

### Supercoiling assay

Reconstituted chromatins consisting of ~50 ng DNA, ~50 ng core histones and chromatin assembly factors, dNAP‐1 (~ 4 ng), dISWI (~ 12 ng) and ATP‐dependent chromatin assembly factor large subunit 1 (dAcf1; ~14 ng), were deprotonated by phenol‐chloroform extraction and EtOH precipitation. Samples were suspended in Milli‐Q water with 0.01 µg·mL^−1^ RNase (Nippon Gene) and separated on a 1.5% agarose gel in TAE buffer and then visualized with ethidium bromide [13, 14].

### MNase assay

Either fresh or freeze‐thawed reconstituted chromatin containing ~25 ng of each plasmid DNA and core histones was digested in the presence of 120 U MNase (TaKaRa, Shiga, Japan) in 20 mm Tris–HCl (pH 8.0), 5 mm NaCl and 2.5 mm CaCl_2_ at 37 ˚C, and the reaction was halted by adding 5 mm EGTA, followed by phenol‐chloroform extraction and EtOH precipitation. Samples were suspended in Milli‐Q water with 0.01 µg·mL^−1^ RNase (Nippon Gene), then separated on a composite gel (2% soft agarose and 0.66% agarose) in TAE buffer and visualized with ethidium bromide staining [13, 14].

### Isolation of chromatin

After chromatin assembly reaction, which contained 2.7 mm magnesium acetate in the translation reaction, reconstituted chromatin mixtures were transferred to 1.5‐mL tubes and diluted with 3 vol of HD buffer (25 mm HEPES, pH 7.8, 1 mm DTT); then MgCl_2_ was added, and the concentration was adjusted as indicated in Fig. S5. In the standard protocol, the assembled chromatin mixture was brought to 10 mm MgCl_2_ and incubated for 5 min at room temperature, followed by centrifugation for 10 min at 10 000 ***g*** at 20 ˚C. The pellet was suspended in HD buffer and directly used as nucleosome samples for further experiments or frozen in liquid nitrogen and stored at −80 ˚C until use.

### Histone modification reaction

*S*‐adenosyl‐l‐methionine (Wako), acetyl coenzyme A (Wako) or ^14^C‐labeled donors, S‐[methyl‐^14^C]‐adenosyl‐l‐methionine (2.146 GBq·mmol^−1^; Perkin Elmer) and [acetyl‐1‐^14^C]‐acetyl coenzyme A (2.22 GBq·mmol^−1^; Perkin Elmer) (each 50 µm containing 7.4 kBq in 30‐µL reaction mixtures), were added to nucleosomes in HD buffer. Modification reactions were started by adding the cell‐free synthesized soluble fractions of unpurified dGCN5 (1.3 pmol) or dSet8 (12.1 pmol), then incubated for 30 min at 26 ˚C. The amount of each enzyme used was adapted from product protocols for purified recombinant human enzymes (Active Motif, Carlsbad, CA, USA). Modified chromatin was precipitated by Mg^2+^, and nucleosomal histones were separated by 15% SDS/PAGE and stained with CBB, or radiolabeled histone bands were visualized by autoradiography.

### Mass spectroscopy

To confirm newly synthesized histones in cell‐free translation, and also to characterize modifications of histones in nucleosomes, we performed mass spectrometry (MS). Proteins were separated by SDS/PAGE and visualized by CBB staining. In‐gel protein digestion was performed as previously described with minor modifications [34]. After SDS/PAGE, gel‐separated histones were digested with sequencing grade trypsin, chymotrypsin, Asp‐N or Arg‐C (Promega, Madison, WI, USA) at an enzyme‐to‐substrate ratio of 1 : 50 (w/w) in 100 mm ammonium bicarbonate buffer, pH 8.8, for overnight at 37 °C. Digested peptides were desalted by self‐made C18 Stage tip and eluted with 40 µL of 0.1% (v/v) TFA/80% (v/v) acetonitrile. Eluates were dried by vacuum centrifugation and redissolved in 10 µL 0.1% (v/v) trifluoroacetic acid (TFA) for MS analysis.

MALDI‐TOF MS analyses were performed by mixing 0.5 µL of peptide solution with an equal amount of matrix solution [2% (w/v) α‐cyano‐4‐hydroxycinnamic acid in 50% (v/v) acetonitrile/0.1% (v/v) TFA] and spotted on a stainless steel MS sample plate. MS spectra were acquired on an AXIMA TOF^2^ MALDI‐TOF mass spectrometer (Shimadzu Corporation, Kyoto, Japan) in the positive ion mode. MS/MS analysis was conducted on a Prominence NanoLC system (Shimadzu) connected to LTQ linear ion trap mass spectrometer (Thermo Electron Fisher, Foster, CA, USA) equipped with a nanoelectrospray ionization source. For the liquid chromatography (LC) separation, mobile phases consisted of 0.1% (v/v) formic acid in H_2_O as solvent A and 0.1% (v/v) formic acid 80% (v/v) in acetonitrile as solvent B. Peptide samples were injected onto a C18 trap column (Chemicals Evaluation and Research Institute, Japan), then separated on a fused‐silica capillary column packed with C18 resin (75‐µm i.d. × 15 cm; Nikkyo Technos, Tokyo, Japan) at a flow rate of 300 nL·min^−1^ in accordance with the following gradient schedule: 10–50 min, 2–30% B; 50–65 min, 30–90% B; hold at 90% B for 10 min, then reequilibrate at 2% B for 15 min. MS/MS spectra were searched against the UniProt *Drosophila* proteome database (download date: June 1, 2013; 41 934 sequences) by the ProteinPilot version 4.0 (SCIEX, Redwood, MA, USA) using the following parameters: cys alkylation, acrylamide; processing parameters, biological modification; and search effort, through ID. The selected reaction monitoring (SRM) analysis was performed using a QTRAP 5500 hybrid triple‐quadrupole/linear ion trap MS (SCIEX) coupled with an Eksigent nanoLC system via a cHiPLC‐nanoflex module (SCIEX) as described previously [34, 35]. The Skyline software was used to build SRM assays [36]. For LC‐SRM assay, targeted peptides were separated on a C18 reversed‐phase cHiPLC column (75‐µm i.d. × 15 cm; SCIEX) at a flow rate of 300 nL/min in accordance with the following gradient schedule: 0–3 min, 2–10% B; 3–30 min, 10–30% B; 30–40 min, 30–90% B; hold at 90% B for 5 min and then reequilibrate at 2% B for 15 min [mobile phase A, 0.1% (v/v) formic acid; mobile phase B, 0.1% (v/v) formic acid/80% (v/v) acetonitrile].

### Computation

*D. melanogaster* histone sequences with UniProt IDs of DmH2A (P84051), DmH2B (P02283), DmH3 (P02299) and DmH4 (P84040) were used [30]. Wheat *Triticum aestivum* histone sequences were also retrieved from UniProt, including TaH2A (A03AB6IP09), TaH2B (W5A7K6), TaH3 (W5GCP8) and TaH4 (A0A3B6U3I5). Pairwise sequence alignment (https://www.ebi.ac.uk/Tools/psa/emboss_needle/) was performed for each histone from two species.

## Results and Discussion

### Synthesis of histones, chromatin assembly factors and histone modification enzymes in the wheat germ cell‐free translation

**Fig. 1:**
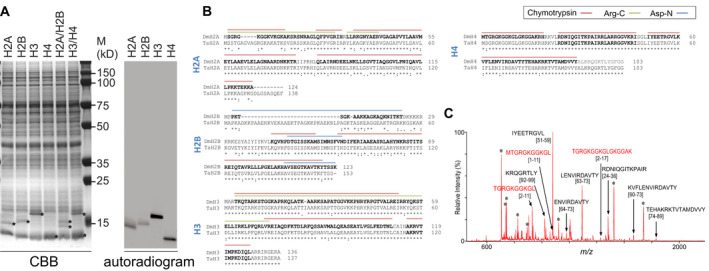
Synthesis of four core histones by wheat germ cell‐free translation and peptide mapping using MS. (A) Each histone was synthesized, and H2A/H2B or H3/H4 were cotranslated by the cup dialysis method at 15 ˚C for 50 h followed by SDS/PAGE and detected by CBB staining and autoradiography. An asterisk (*) denotes each product with expected molecular mass (*m*). (B) Protein sequence alignment of *Drosophila* and wheat (*Triticum aestivum*) histones H2A, H2B, H3 and H4 is shown with the sequences (bold) assigned by LC‐MS/MS analysis with different proteases as indicated. The processing of N‐terminal methionine was observed in all histones, while a peptide fragment containing N‐terminal methionine was observed slightly in histone H4. In our database search for obtained MS/MS spectra, no chemical modification was identified. Asterisk (*), colon (:) and dot (.) denote perfect alignment, amino acid residue with strong similarity and weak similarity, respectively. (C) Further evaluation of the N‐terminal processing of histone H4 by the MALDI‐TOF MS. Although a partially unprocessed N‐terminal chymotryptic peptide, MTGRGKGGKGL, was observed, N‐terminal methionine excision was predominantly observed in this analysis. Asterisks (*) indicate the peptide ions assigned to chymotrypsin autolysis products.

To produce chromatin *in vitro* using the wheat germ cell‐free protein synthesis, we first attempted to synthesize the following *Drosophila* proteins: histones H2A, H2B, H3 and H4; histone chaperone, dNAP‐1; ATP‐dependent chromatin remodeling factors, dAcf1 and dISWI; and chromatin‐modifying enzymes, dGCN5 and dSet8. pEU plasmids harboring the earlier genes were used as templates for the SP6 RNA polymerase to generate the mRNAs *in vitro*. Synthesized mRNAs were treated with DNase I to remove residual pEU plasmid and then used for *in vitro* wheat germ cell‐free translation to produce *Drosophila* proteins for 50 h at 15 ˚C in dialysis mode. The *in vitro*‐translated products were analyzed by SDS/PAGE, followed by CBB staining or autoradiography (Figs 1 and S1). The four core histones of the correct size can be seen among the wheat germ extract proteins. Autoradiograms show that all products were intact and of the expected molecular weight except for dSet8, which was larger than the expected size of 76 kDa and will be discussed later. The calculated concentration (pmol·µL^−1^) and solubility (%) from at least three independent experiments of individually synthesized histones H2A, H2B, H3 and H4 were 13.8 ± 2.8, 14.4 ± 1.4, 16.2 ± 0.7 and 15.5 ± 1.4 pmol·µL^−1^ and 56% ± 5%, 60% ± 2%, 51% ± 5% and 59% ± 4%, respectively. Similarly, concentration (pmol·µL^−1^) and solubility (%) of dNAP‐1, dAcf1, dISWI and histone‐modifying enzymes, dGCN5 and dSet8, were 24.8 ± 6.8, 3.6 ± 0.7, 2.7 ± 0.3, 15.9 ± 1.0 and 11.1 ± 0.8 pmol·µL^−1^ and 100% ± 1%, 12% ± 1%, 32% ± 6%, 11% ± 1% and 21% ± 2%, respectively. The observed solubility of four core histones was greater than 50%, which enabled the immediate use of those products for chromatin assembly *in vitro*.

After *in vitro* translation, products were analyzed by MS, and it was confirmed that synthesized histones were N‐terminal methionine processed but otherwise lacked any posttranslational modification (Fig. 1B,C; Table S1). As shown in Fig. 1B, both *Drosophila* and wheat histone sequences were aligned, and the two peptide fragments from each of histones H3 (Ala115–Leu127) and H4 (Arg24–Ile47) were determined to be identical. However, other wheat histone derivatives were not identified by MS/MS analysis. It is also important to note that we did not detect any endogenous wheat histone in the translation mixture in the previous MS analysis [30]. These results provide an important advantage to using the wheat cell‐free synthesized histones over heterogeneously modified histones derived from cell nuclei. Furthermore, a major fraction of the synthesized histones remained soluble and had not aggregated, which provides a great advantage over conventional methods that use recombinant histones.

To develop a streamlined method for chromatin assembly, we coexpressed histones H2A and H2B (H2A/H2B), as well as H3 and H4 (H3/H4), and we noticed that H3/H4 resulted in a 1.2‐fold molar yield compared with H2A/H2B (Fig. 1A). To understand the observed higher productivity of the coexpressed H3/H4 over H2A/H2B, we tested the kinetics of H2A/H2B and H3/H4 coexpression reactions in the presence of ^14^C‐leucine using the conventional batch‐wise wheat germ translation at 26 ˚C (Fig. S2A). The result showed that incorporation of ^14^C‐leucine into both H2A/H2B and H3/H4 ceased after 2 h, which was noticeably short because many target proteins can be continuously synthesized up to 20 h in wheat cell‐free translation [32, 33, 37]. This lag of histone synthesis led us to hypothesize that those newly synthesized highly basic histones might interact with mRNAs or other factors in the wheat translation apparatus, thereby interfering with the translation process. These results are consistent with the observation that free histones bind RNA, inhibiting translation [38]. To reduce the amount of free histones in the reaction, we added plasmid DNA to the reaction after 2 h of incubation, which resulted in the reinitiation of translation, although this effect was seen in only the H3/H4 coexpression and not in H2A/H2B coexpression (Fig. S2A,B). The observed improvement was thus likely due to coexpression and immediate assembly of H3/H4 with pBS during the synthesis.

### Posttranslational nucleosome assembly

Having developed conditions to efficiently synthesize core histones and chromatin assembly factors using the wheat germ cell‐free system, we investigated the ability of *in vitro* chromatin assembly. We first examined whether the wheat germ extract contained endonuclease activity that could degrade plasmid DNA, because this was not tested in a previous study [30]. SC‐pBS was incubated in the translation mixture at 26 ˚C (Fig. S4), and results showed no detectable level of DNase activity in the extract even after prolonged incubation. Interestingly, the SC‐pBS was converted to the relaxed form of pBS within 10 min of incubation (Fig. S3). This result indicated that there is endogenous topoisomerase activity in the wheat germ extract. The observed topoisomerase activity was inhibited by 50 µm topoisomerase I‐specific inhibitor (camptothecin), but not by topoisomerase II‐specific inhibitor (ICRF‐193). Thus, the observed topoisomerase activity was concluded to be mediated by endogenous topoisomerase type I activity in the wheat extract. This result enabled us to measure the efficiency of chromatin assembly using the extent of supercoiling as a readout for nucleosome deposition [39] without supplementing exogenous topoisomerase.

**Fig. 2:**
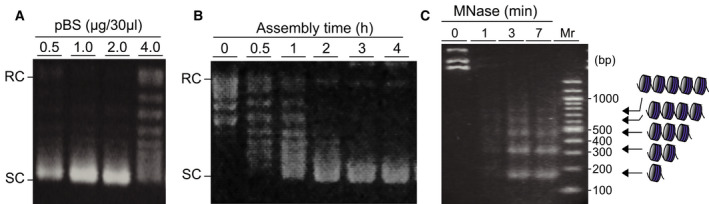
The results from supercoiling assay and MNase assay on the reconstituted chromatin. (A) Different amounts of pBS were tested with a constant amount of histones in the presence of three chromatin assembly factors, dNAP‐1, dAcf1 and dISWI. (B) Time courses of the chromatin assembly reaction. (C) MNase assay for reconstituted chromatin with indicated time. RC and SC on agarose gel indicate nicked relaxed pBS and SC‐pBS, respectively. Mr is a 100‐bp DNA marker. Gel images show representative examples of three replicates.

To optimize the chromatin assembly reaction, we first determined the optimum ratio of individually synthesized four core histones to template DNA in the reaction containing histone chaperone and ATP‐dependent chromatin assembly factors, dNAP‐1, dAcf1 and dISWI (Fig. 2A). It was determined that 1.0–2.0 µg (1.1–2.2 pmol) of pBS plasmid was the most effective when mixed with 1.0 µg (20 pmol of each core histone) of total core histones in the 30 µL assembly reaction, consistent with previous studies [7, 40], whereas 4.0 µg (4.4 pmol) of pBS plasmid to 1.0 µg core histones reduced the formation of supercoiling. The reaction time was also tested, and we found that a 3‐h assembly reaction is sufficient for completion (Fig. 2B), similar to the *Drosophila* chromatin assembly system reported earlier [7]. In the coexpression‐mediated chromatin assembly shown in a previous study [30], the translation‐coupled reconstitution took ~6 h, whereas the current method does not require a translation process, which differs between two reconstitution modes. Determined conditions were used as standard assembly reactions for the following experiments. In Fig. 2C, we evaluated the nucleosome repeat length in chromatin assembled on pBS under optimized reaction conditions. After chromatin assembly, MNase was added to samples, and a time course of digestion was conducted. Digested DNA was analyzed by agarose gel electrophoresis; then nucleosome repeat length was estimated. The average repeat length was around 165 bp, indicating that histones had indeed been successfully incorporated into nucleosomes and formed chromatin on the DNA.

**Fig. 3:**
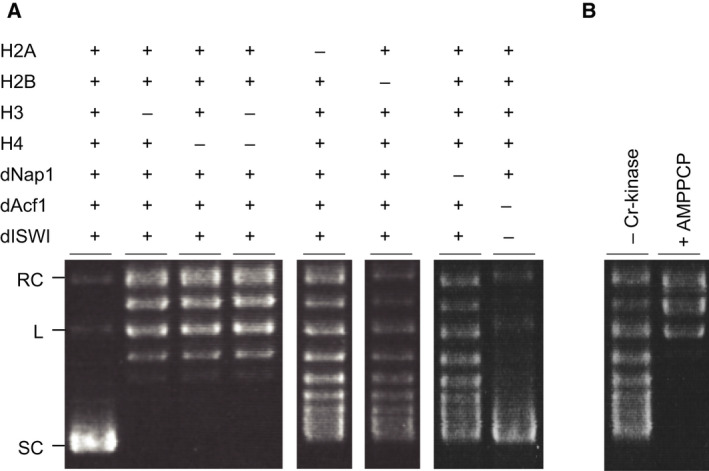
Essential components for chromatin assembly. (A) Supercoiling assay for assembled chromatin by reactions in the presence (+) and absence (−) of individually synthesized histones and chromatin assembly factors, dNAP‐1, dAcf1 and dISWI. (B) Supercoiling assay in the absence of creatine kinase (−Cr‐kinase) and presence of ATP analogue (+AMP‐PCP). Gel images show representative examples of three replicates. These results in the full‐length gel are provided in Fig. S4. L, for linear plasmid; RC, relaxed circular plasmid; SC, supercoiled plasmid.

To determine which component is essential for the chromatin assembly, we eliminated each component and assessed by supercoiling assay (Figs 3 and S4). In the absence of either histone H3 or H4, or both H3/H4, the formation of supercoils was not detected (Figs 3A and S4A). When either histone H2A or H2B was absent, the supercoiling formation was still observed, but to a lesser extent than with the complete reaction. Consistent with the previous reports, histones H3 and H4 are essential, whereas in the absence of H2A or H2B, supercoils could still be stabilized by the H3/H4 tetramer [11]. We also tested the requirements of the dNAP‐1 and ACF (dAcf1 and dISWI) by supercoiling assay [7]. In the absence of dNAP‐1, the extent of supercoiled DNA was reduced compared with the complete assembly reaction. When lacking ACF (dAcf1 and dISWI), the amount of supercoiled DNA was slightly diminished. These results differ from previous studies, in which both dNAP‐1 and ACF have been shown to be essential in the supercoiling formation [21, 41, 42]. It is thought that there are possible chromatin assembly factors present in the wheat germ extract, which were not found in the proteomic analysis in the previous study [30]. Because ACF aids chromatin assembly in an ATP‐dependent manner, we next assessed the effect of ATP on chromatin assembly by using a nonhydrolyzable ATP analogue, AMP‐PCP (Figs 3B and S4B). As a result, the ATP‐regenerating system was essential for the formation of chromatin, indicating that there are alternative ATP‐dependent chromatin assembly factors that help the formation of chromatin in our system. In contrast, the previously described coexpression reconstitution reaction contained an ATP‐regenerating system required for protein synthesis; thus, the requirement of ATP in the chromatin assembly reaction was not assessed [30]. Although we do not know what chromatin assembly factors present in the extract, it is interesting to determine them in the future study.

### Isolation of the reconstituted chromatin

Conventionally, *in vitro* assembled or native chromatin is purified by sucrose density gradient or size exclusion chromatography, both of which require tens of micrograms of the sample caused by losses during these processes [43]. The effect of divalent or multivalent cations on DNA–protein complex condensation had been described in previous studies [44, 45, 46]. We therefore decided to test the ability of Mg^2+^ to condense and isolate assembled chromatin from a crude assembly mixture. Increasing amounts of Mg^2+^ were added to the extract, followed by brief centrifugation to fractionate the reaction into supernatant and pellet (Fig. S5A). At 2.7 mm Mg^2+^, which is the concentration included in the standard wheat translation reaction, most pBS was still found in the supernatant. At 6 mm Mg^2+^, nearly equal amounts of DNA were found in both fractions. At 10 mm Mg^2+^, most of pBS was found in the pellet; thus, we used this concentration throughout the Mg^2+^ precipitation of chromatin. In addition, in the presence of histones H3 and H4, pBS was found predominantly in the pellet (Fig. S5B), suggesting that H3/H4 and pBS formed a complex. In contrast, we did not find any pBS in the pellet in the absence of histones. As a control, *E. coli* ribosomal protein S7, which is a similarly small and basic protein as histones, was tested at 10 mm Mg^2+^, and no pBS was found in the precipitant (Fig. S5B, lane S7). Thus, the precipitated DNA is likely a chromatinized form. We also determined whether Mg^2+^‐dependent precipitation was specific for DNA–protein complexes, or whether Mg^2+^ could also precipitate RNA–protein complexes. The supernatant and pellet from the samples containing wheat ribosome without RNase treatment were analyzed on an agarose gel (Fig. S5B, lane rRNA). This result shows that a mixture of both ribosomes and tRNAs was found exclusively in the supernatant, while the pBS was found only in the pellet, indicating that the effect of Mg^2+^ was specific on the histone–DNA complexes (Fig. S5B). The same samples were subjected to SDS/PAGE analysis to confirm the existence of nucleosomal histones in the Mg^2+^‐isolated fraction (Fig. S5C). The majority of wheat germ proteins were found in the supernatant, while histones H2A, H2B, H3 and H4 were found predominantly in the pellet at 10 mm Mg^2+^. Thus, the assembled chromatin was isolated by the use of 10 mm Mg^2+^ and subsequent centrifugation. In addition, we were able to isolate histones H3 and H4 with pBS, indicative of the formation of histone H3/H4 tetramers in the assembly (Fig. S5C). Moreover, histones were not isolated when the reaction was carried out in the presence of AMP‐PCP, proving that ATP hydrolysis is essential for chromatin assembly.

To ask whether the molar ratios of four histones in the pellet retained the stoichiometry in chromatin before and after Mg^2+^ isolation, we performed the chromatin assembly with radioactively labeled histones, fractionated using 10 mm Mg^2+^, and separated histones on SDS/PAGE. The amount of radioactivity in each histone was measured and standardized to the number of leucine residues present in each molecule. These results show that isolated complexes were composed of four histones with close to equimolar ratios (Fig. S6).

An additional advantage of the described chromatin assembly and isolation method is that chromatin could be stored stably at −80 ˚C. In Fig. S7, we examined the stability of an assembled chromatin upon repeated freezing and thawing by MNase digestion. The digestion profiles of this chromatin before and after four cycles of freezing and thawing were indistinguishable (Fig. S7B), although unfractionated chromatin led to a loss of the MNase pattern likely because of aggregation (Fig. S7A, compared with the freshly prepared sample in Fig. 2C). The observed reversible chromatin isolation by Mg^2+^ in our method might also be an effect of currently unknown endogenous proteins in the wheat extract, and they will need to be determined in future studies.

### A modification of reconstituted chromatin by histone modifiers

**Fig. 4:**
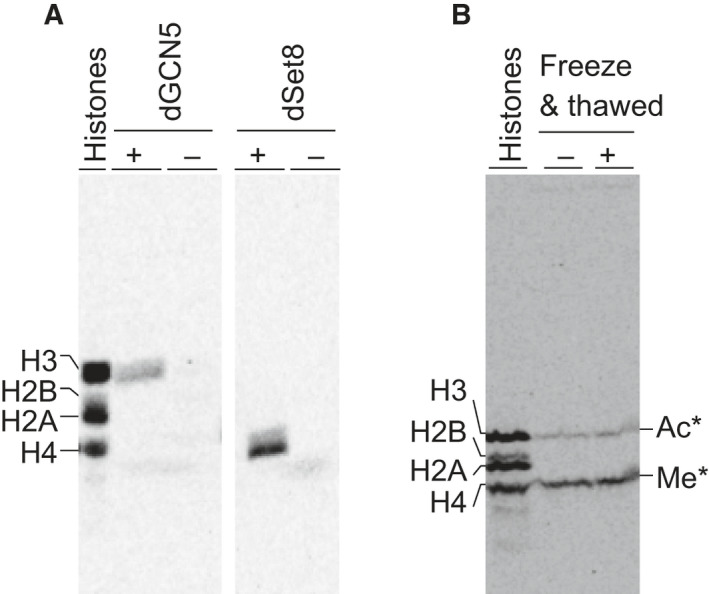
Modification of nucleosomal histones by histone modification enzymes, dGCN5 and dSet8. (A) Mg^2+^‐isolated nucleosomes from standard chromatin assembly reaction were suspended in HD buffer and incubated with dGCN5 or dSet8 in the presence of radiolabeled SAM or acetyl‐CoA, then analyzed by autoradiography. (B) Similar analyses of the modification were performed in the presence of both dGCN5 and dSet8 together with donors on those isolated chromatins before and after freezing and thawing samples. The ^14^C‐Leu‐labeled four histones were used as markers, and dots marked each histone, H3, H2B, H2A and H4, from the top to bottom. Gel images show representative examples of three replicates.

We next asked whether assembled chromatin could serve as a substrate for histone‐modifying enzymes, for the first time on the basis of nonchemically modified ATP‐dependent assembled chromatin. dGCN5 is a histone acetyltransferase that acetylates the N‐terminal tail of both free and nucleosomal histone H3. This activity is aided by other factors, such as dADAs [47, 48]. Histone methyltransferase, dSet8, is known to methylate the N‐terminal tail of histone H4 [49, 50]. The Mg^2+^‐isolated chromatin in HD buffer was subjected to histone modification reaction by adding *in vitro*‐synthesized dGCN5 or dSet8 in the presence of radiolabeled acetyl‐CoA or *S*‐adenosyl methionine, respectively (Fig. 4A). Acetylation of histone H3 by dGCN5 and methylation of histone H4 by dSet8 were monitored by autoradiography, respectively. Results showed that histones were modified in an enzyme‐dependent manner, demonstrating that isolated chromatin can serve as a substrate for these histone‐modifying enzymes. The freeze‐thawed chromatin was also tested in this assay, and we found that repeated freeze‐thawed chromatin was also modified by both enzymes (Fig. 4B).

The sites of modification on the histones H3 and H4 were determined by MS (Figs S8 and S9). This analysis showed that the H3 peptide (Ser10–Lys23) was acetylated at both the K14 and K18 positions by dGCN5, which is consistent with previous studies [50]. Furthermore, monomethylation of histone H4 K20 by dSet8 was confirmed by MALDI‐TOF MS, as was reported earlier [49]. Notably, the discrepancy between the observed molecular weight of cell‐free synthesized dSet8 and the expected size has been described in an earlier *in vivo* study [49]. Thus, we provide evidence that both Mg^2+^‐isolated and freeze‐thawed chromatin maintained a structure that histone‐modifying enzymes could recognize as their substrates, and hence it was also concluded that some residual proteins after Mg^2+^ isolation (Fig. S5) do not inhibit subsequent chromatin modification reaction.

### Comparison of chromatin assembly using wheat germ cell‐free synthesis and conventional methods

We have described a method to synthesize histones and reconstitute chromatin by using wheat germ cell‐free synthesis. Table 1 presents a comparison of different methods available for *in vitro* chromatin assembly to date. The salt dialysis method requires either recombinantly expressed histones or extracted core histones from living tissues [12, 15, 43]. Although recombinantly expressed histones do not possess any posttranslational modification, they need to be refolded, and their ability to be incorporated into chromatin is limited [7, 12]. The use of native histones from cells and tissues overcomes these drawbacks, although they are a heterogeneous mixture with various histone subtypes and posttranslational modifications that altogether might confound the downstream analyses [7, 8, 12, 28]. Furthermore, extracted histones from living tissues need high‐salt or acid treatment during the purification depending on the source of the histones [51]. Finally, the nucleosomes assembled with salt dialysis are not uniformly spaced unless the underlying DNA template has a repeating array of a defined sequence such as the 601 repeat [17, 18, 19]. The latter problem can be overcome using histone chaperones and ATP‐dependent chromatin remodeling factors [7]. Finally, chromatin assembled with conventional methods needs to be stored at 4 °C and is stable for a few months, whereas Mg^2+^‐isolated chromatin from our method was shown to be stable over several repeats of freeze‐thawing and can be stored stably over several months at −80 °C.

**Table 1:** Comparison of *in vitro* chromatin reconstitution methods.

*In vitro* assembly methods	Source histones	State of histones	PTMs^a^	Storage^b^	References
Salt dialysis (recombinant histones)	Any	Need refolding	None	Approximately a few months at 4 ˚C	[12, 15, 43]
Salt dialysis (extracted histones)	Cell nuclei origin	Native (high‐salt extraction)	Present	Approximately a few months at 4 ˚C	[12, 15, 43]
Chaperone‐mediated methods (*Escherichia coli* recombinant)	Any	Need refolding	None	Approximately a few months at 4 ˚C	[7, 14]
Wheat cell‐free synthesis‐based posttranslational chromatin assembly	Any	Native	None	Over several months at 4 ˚C after Mg^2+^ isolation	[30], this study

^a^ Presence or absence of the posttranslational modifications (PTMs) of histones.

^b^ Reported chromatin storage conditions.

In our wheat cell‐free chromatin assembly method, the source of histones might be any organism of interest, and histones can be synthesized in a native state. Moreover, these histones are free of any detectable posttranslational modification; thus, chromatin reconstitution can be performed on any circular DNA molecule.

## Conflict of interest

The authors declare no conflict of interest.

## Author contributions

YE and TET conceived and designed the experiments, and YE, NT and KO performed the experiments. YE, NT, SKN, KO, RK, HO and TET wrote the manuscript.

## Supporting information


**Fig. S1**. Proteins synthesized in cell‐free translation. Autoradiograms of synthesized ATP‐dependent chromatin remodeling factors, dAcf1, dISWI, and histone chaperone, dNap‐1 (A), and histone modifiers dGCN5 and dSet8 (B) are shown. Star denotes each product with expected molecular weight.
**Fig. S2**. Cotranslation of histones in the batch and bilayer translation reactions. (A) An equal amount of each of mRNA for cotranslation of two histones H2A/H2B (filled square) and H3/H4 (filled circle) were performed in batch‐wise translation. Translation reaction was initiated without pBS, and 2 h after when translation appeared to be ceased, pBS was added at final concentration of 40 μg·mL^−1^ marked with arrows. Open square and circle indicate histone H2A/H2B and H3/H4 after addition of pBS, respectively. Synthesis of histones was monitored by ^14^C‐leucine incorporation into a hot‐TCA insoluble fraction. (B) Autoradiograms of the synthesized histones H3 and H4 products. The bilayer translation containing 5 μg of mRNAs for histones H3 and H4 in the absence or presence of 1.0 μg of pBS in 20 μL of the bottom layer are shown.
**Fig. S3**. Topoisomerase activity was determined in the wheat germ extract. The mixture (60 μL) contained 10 μL of stock wheat germ extract (final 40 *A*_260_/mL), creatine kinase (40 U·mL^−1^) and 6 μg of SC‐pBS in the absence of histones or chaperones incubated at 26 ˚C for the indicated time.
**Fig. S4**. Supplementary gel image for the results of supercoiling assay shown in Fig. 3. (A) Supercoiling assay for assembled chromatin by reactions in the presence (+) and absence (‐) of individually synthesized histones and chromatin assembly factors are shown in the full‐length gel. (B) Effect of the absence of creatine kinase or presence of AMP‐PCP on supercoiling formation is shown in the full‐length gel.
**Fig. S5**. Isolation of histone–DNA complexes with the use of Mg^2+^. (A) The final concentrations at 2.7, 6.0 and 10.0 mm MgCl2 were used after completion of chromatin assembly reaction to isolate histone–DNA complexes followed by the centrifugation. Phenol‐extracted plasmid from the total reaction (T), supernatant (S) and pellet (P) was run on agarose gel. Nicked relaxed, linear and supercoiled pBS are indicated as RC, L and SC, respectively. (B) The cotranslated H3/H4 or ribosomal protein S7 was incubated in the presence of pBS. After the addition of 10 mm MgCl2, samples were separated into S and P fractions, and pBSs were separated. In the rRNA lanes, both S and P loading samples were prepared from RNase‐untreated complete assembled mixture as in (A) with 10 mm MgCl2. (C) Assembled mixtures with four core histones or with H3/H4 were separated into two fractions, S and P, with 10 mm MgCl2 and then run on SDS/PAGE. Also, a similar experiment in complete assembly reaction was performed in the presence of 1 mm AMPPCP. Amounts of DNA or proteins loaded among lanes were adjusted to an equivalent volume of assembly reaction mixtures. Recovery of proteins in Mg2+‐precipitants and purity of histones were calculated by comparison of total intensities of CBB‐stained bands to ones of Mg2+‐precipitates, or core histones in Mg2+‐precipitates by densitometry. Arrows indicate the positions of wheat endogenous nonhistone proteins near histones H3 and H4. Gel images show representative examples of at least three replicates.
**Fig. S6**. Stoichiometry of histones. Autoradiograms of core histones in the mixture (A, total) and in the Mg2+‐isolated sample (B) of PNAP assembled chromatin. The intensity of each histone band was measured by the densitometry, and the relative molar ratios of histones H2A, H2B and H4 to H3 were calculated as described in the Materials and Methods and shown on the right.
**Fig. S7**. Effect of freezing and thawing on the purified chromatin. Samples of the assembled reaction mixture or Mg2+‐isolated chromatin in the HD buffer were flash frozen by the liquid nitrogen and thawed at room temperature. MNase assay was performed for once freeze‐dried unpurified chromatin (A), Mg2+‐isolated chromatin (B, left) and four times freezing and thawing Mg2+‐isolated chromatin (B, right). The number of nucleosome arrays is indicated with the arrow.
**Fig. S8**. Selected reaction monitoring (SRM) analysis of *in vitro* histone acetylation by dGCN5. The presence of acetylated lysine residues in the dGCN5‐treated histone sample was verified using the LC‐SRM analysis, which enables selective detection of the targeted peptides from crude samples. Based on the MS/MS information obtained from the tryptic peptides of acetylated histone H3, we selected a set of the fragment ions (Q3) for each precursor ion (Q1) and finally established the SRM assays. The SRM analysis coupled with LC separation followed by confirmation of the chromatographic profiles of the targeted peptides was performed. Two acetylated peptides, STGGKAPRKQLATK [10‐23 amino acids (a.a.)] and APRKQLATK [15‐23 a.a.], were determined only in the dGCN5‐treated sample. For the peptide STELLIRK [57‐64 a.a.] with no reported modification by dGCN5, no difference was observed with or without dGCN5 treatment. K* denotes the acetylated lysine; Q* denotes the deaminated glutamine.
**Fig. S9**. MALDI‐TOF MS analysis of *in vitro* histone methylation by dSET8. (A) After digestion with chymotrypsin, derived digests were subjected to peptide mass fingerprinting (PMF) analysis using the MALDI‐TOF mass spectrometer. Asterisk indicates the peptide ions assigned chymotrypsin autolysis products. (B) PMF change by the dSET8 treatment was observed in the range of *m/z* 1250‐1350. The ion peaks at 1306.8 and 1320.8 exhibit a mass difference of 14 Da, a predicted mass shift given by the monomethylation, indicating the presence of lysine methylation by dSET8 in histone peptide GKGGAKRHRKVL.

**Table S1**. Peptide mapping of *Drosophila* histones by LC‐MS/MS analysis.

## Data Availability

The data that support the findings of this study are available from the corresponding author (takasuka@cen.agr.hokudai.ac.jp) upon reasonable request.
