# Supplementary material for: De novo reconstitution of chromatin using wheat germ cell‐free protein synthesis
Source: FEBS Open Bio. 2021 May 16;11(6):1552–64. doi: 10.1002/2211-5463.13178 (PMC8167859; doi:10.1002/2211-5463.13178)
Supplement: Supplementary file 1 — Fig. S1. Proteins synthesized in cell‐free translation. Autoradiograms of synthesized ATP‐dependent chromatin remodeling factors, dAcf1, dISWI, and histone chaperone, dNap‐1 (A), and histone modifiers dGCN5 and dSet8 (B) are shown. Star denotes each product with expected molecular weight. Fig. S2. Cotranslation of histones in the batch and bilayer translation reactions. (A) An equal amount of each of mRNA for cotranslation of two histones H2A/H2B (filled square) and H3/H4 (filled circle) were performed in batch‐wise translation. Translation reaction was initiated without pBS, and 2 h after when translation appeared to be ceased, pBS was added at final concentration of 40 μg·mL−1 marked with arrows. Open square and circle indicate histone H2A/H2B and H3/H4 after addition of pBS, respectively. Synthesis of histones was monitored by 14C‐leucine incorporation into a hot‐TCA insoluble fraction. (B) Autoradiograms of the synthesized histones H3 and H4 products. The bilayer translation containing 5 μg of mRNAs for histones H3 and H4 in the absence or presence of 1.0 μg of pBS in 20 μL of the bottom layer are shown. Fig. S3. Topoisomerase activity was determined in the wheat germ extract. The mixture (60 μL) contained 10 μL of stock wheat germ extract (final 40 A 260/mL), creatine kinase (40 U·mL−1) and 6 μg of SC‐pBS in the absence of histones or chaperones incubated at 26 ˚C for the indicated time. Fig. S4. Supplementary gel image for the results of supercoiling assay shown in Fig. 3. (A) Supercoiling assay for assembled chromatin by reactions in the presence (+) and absence (‐) of individually synthesized histones and chromatin assembly factors are shown in the full‐length gel. (B) Effect of the absence of creatine kinase or presence of AMP‐PCP on supercoiling formation is shown in the full‐length gel. Fig. S5. Isolation of histone–DNA complexes with the use of Mg2+. (A) The final concentrations at 2.7, 6.0 and 10.0 mm MgCl2 were used after completion of chromatin assemb [file FEB4-11-1552-s001.pdf]

**SUPPORTING INFORMATION**

# *De novo* reconstitution of chromatin using the wheat cell-free protein synthesis

**Yaeta Endo [1], Nobuaki Takemori [2], Szilvia K. Nagy [3,4], Kei-ichi Okimune [4], Rohinton Kamakaka [5], Hitoshi Onouchi [4], and Taichi E. Takasuka [4,6*]**

Running title: *In vitro wheat cell-free chromatin assembly*

[1] Proteo-Science Center of Ehime University, Matsuyama 790-8577, Japan

[2] Division of Analytical Bio-Medicine, Advanced Research Support Center, Ehime University, Toon 791-0295, Japan

[3] Department of Molecular Biology, Institute of Biochemistry and Molecular Biology, Semmelweis University, Budapest, Hungary

[4] Research Faculty of Agriculture, Hokkaido University, Sapporo 060-8589, Japan

[5] Department of Molecular Cell and Developmental Biology, University of California at Santa Cruz, U.S.A.

[6] Global Institute for Collaborative Research and Education, Hokkaido University, Sapporo 060-8589, Japan

*To whom correspondence should be addressed: **Taichi E. Takasuka**: Research Faculty of Agriculture, Hokkaido University, Sapporo 060-8589, Japan: takasuka@cen.agr.hokudai.ac.jp

**Figure S1:** Proteins synthesized in cell-free translation. Autoradiograms of synthesized ATP-dependent chromatin remodeling factors, dAcf1, dISWI, and histone chaperone, dNap-1 **(a)**, and histone modifiers dGCN5 and dSet8 **(b)** are shown. Star denotes each product with expected molecular weight.

**Figure S2:** Co-translation of histones in the batch and bilayer translation reactions. **(a)** An equal amount of each of mRNA for co-translation of two histones H2A/H2B (filled square), and H3/H4 (filled circle) were performed in batch wise translation. Translation reaction was initiated without pBS, and 2 h after when translation appeared to be ceased, pBS was added at final concentration of 40 μg/mL marked with arrows. Open square and circle indicate histone H2A/H2B and H3/H4 after addition of pBS, respectively. Synthesis of histones was monitored by $^{14}$C-leucine incorporation into a hot-TCA insoluble fraction. **(b)** Autoradiograms of the synthesized histones H3 and H4 products. The bilayer translation containing 5 μg of mRNAs for histones, H3 and H4 in the absence or presence of 1.0 μg of pBS in 20μL of the bottom layer are shown.

**Figure S3:** Topoisomerase activity was determined in the wheat germ extract. The mixture (60 μL) contained 10 μL of stock wheat germ extract (final 40 $A_{260}$/mL), creatine kinase (40 U/mL), and 6 μg of SC-pBS in the absence of histones nor chaperones were incubated at 26˚C for the indicated time.

**Figure S4:** Supplementary gel image for the results of supercoiling assay shown in Fig. 3. **(a)** Supercoiling assay for assembled chromatin by reactions in the presence (+) and absence (-) of individually synthesized histones and chromatin assembly factors are shown in the full-length gel. **(b)** Effect of the absence of creatine kinase or presence of AMPPCP on supercoiling formation is shown in the full-length gel.

**Figure S5.** Isolation of histone-DNA complexes with the use of $Mg^{2+}$. **(a)** The final concentrations at 2.7, 6.0, and 10.0 mM $MgCl_2$ were used after completion of chromatin assembly reaction to isolate histone-DNA complexes followed by the centrifugation. Phenol-extracted plasmid from the total reaction (T), supernatant (S), and pellet (P) was run on agarose gel. Nicked relaxed, linear and supercoiled pBS are indicated as RC, L, and SC, respectively. **(b)** The co-translated H3/H4 or ribosomal protein S7 was incubated in the presence of pBS. After the addition of 10 mM $MgCl_2$, samples were separated into S and P fractions, and pBS were separated. In the rRNA lanes, both S and P loading samples were prepared from RNase-untreated complete assembled mixture as in **(a)** with 10 mM $MgCl_2$. **(c)** Assembled mixtures with four core histones, or with H3/H4 were separated into two fractions, S and P with 10 mM $MgCl_2$ then run on SDS-PAGE. Also, a similar experiment in complete assembly reaction was performed in the presence of 1 mM AMPPCP. Amounts of DNA or proteins loaded among lanes were adjusted to an equivalent volume of assembly reaction mixtures. Recovery of proteins in $Mg^{2+}$-precipitants and purity of histones were calculated by comparison of total intensities of CBB-stained bands to ones of $Mg^{2+}$-precipitates, or core histones in $Mg^{2+}$-precipitates by densitometry. Arrows indicate the positions of wheat endogenous non-histone proteins near histones H3 and H4. Gel images show representative examples of at least 3 replicates.

**Figure S6:** Stoichiometry of histones. Autoradiograms of core histones in the mixture (**a**, total) and in the $Mg^{2+}$-isolated sample **(b)** of PNAP assembled chromatin. The intensity of each histone band was measured by the densitometry, and the relative molar ratios of histones H2A, H2B and H4 to H3 were calculated as described in the Materials and Methods and shown on the right.

**Figure S7:** Effect of freezing and thawing on the purified chromatin. Samples of the assembled reaction mixture or $Mg^{2+}$-isolated chromatin in the HD buffer were flash frozen by the liquid nitrogen and thawed at room temperature. MNase assay was performed for once freeze-dried unpurified chromatin **(a)**, $Mg^{2+}$-isolated chromatin **(b**, left**)**, and 4 times freezing and thawing $Mg^{2+}$-isolated chromatin **(b**, right**)**. The number of nucleosome arrays is indicated with the arrow.

**Figure S8:** Selected reaction monitoring (SRM) analysis of in vitro histone acetylation by dGCN5. The presence of acetylated lysine residues in dGCN5-treated histone sample was verified using the LC-SRM analysis, which enables selective detection of the targeted peptides from crude samples. Based on the MS/MS information obtained from the tryptic peptides of acetylated histone H3, we selected a set of the fragment ions (Q3) for each precursor ion (Q1) and finally established the SRM assays. The SRM analysis coupled with LC separation followed by confirmation of the chromatographic profiles of the targeted peptides was performed. Two acetylated peptides, STGGKAPRKQLATK [10-23 a.a.] and APRKQLATK [15-23 a.a.], were determined only in the dGCN5-treated sample. For the peptide STELLIRK [57-64 a.a.] with no reported modification by dGCN5, no difference was observed with or without dGCN5 treatment. K* denotes the acetylated lysine and Q* denotes the deaminated glutamine, respectively.

**Figure S9:** MALDI-TOF MS analysis of in vitro histone methylation by dSET8. **(a)** After digestion with chymotrypsin, derived digests were subjected to peptide mass fingerprinting (PMF) analysis using the MALDI-TOF mass spectrometer. Asterisk indicates the peptide ions assigned chymotrypsin autolysis products. **(b)** PMF change by the dSET8 treatment was observed in the range of m/z 1250-1350. The ion peaks at 1306.8 and 1320.8 exhibit a mass difference of 14 Da, a predicted mass shift given by the mono-methylation, indicating the presence of lysine methylation by dSET8 in histone peptide GKGGAKRHRKVL.

**Figure S1**

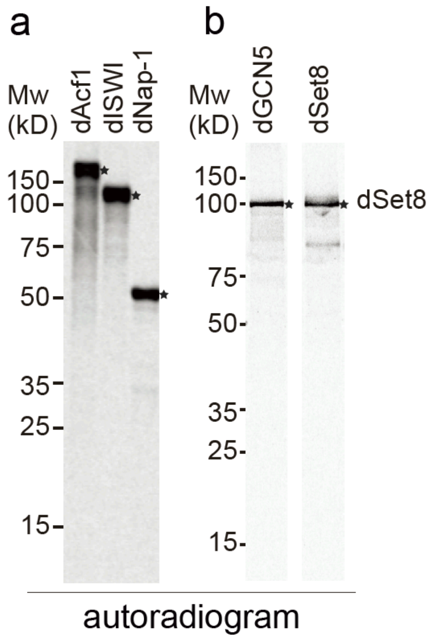
a
b
Mw
(kD)
dAcf1
dISWI
dNap-1
150
100
75
50
35
25
15
Mw
(kD)
dGCN5
dSet8
150
100
75
50
35
25
15
dSet8
autoradiogram

**Figure S2**

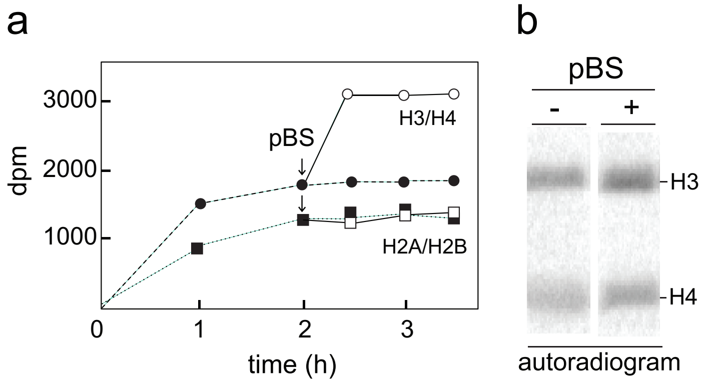
a
3000
2000
1000
dpm
0
1
2
3
time (h)
pBS
H3/H4
H2A/H2B
b
pBS
-
+
H3
H4
autoradiogram

**Figure S3**

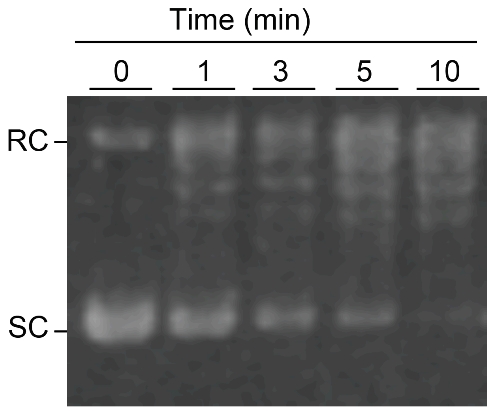

**Figure S4**

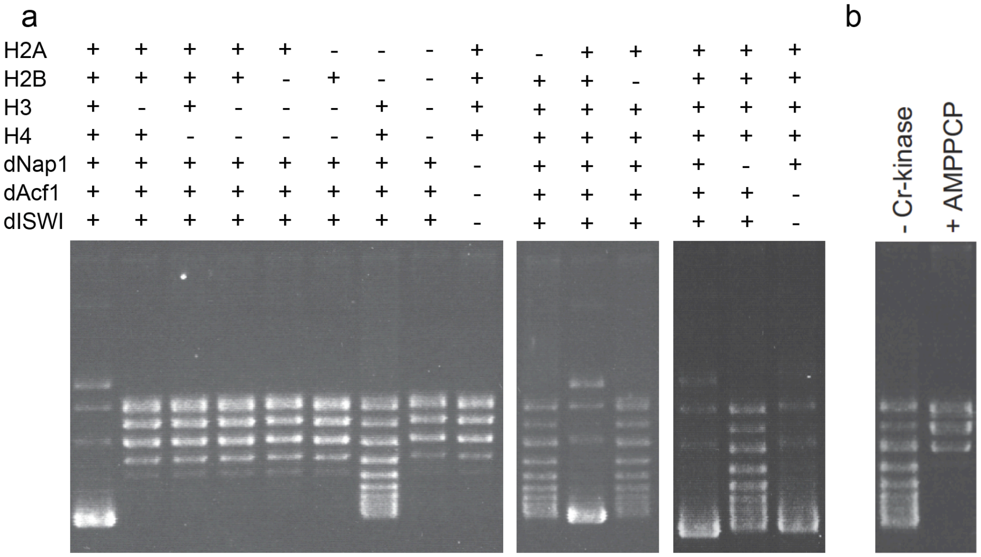

**Figure S5**

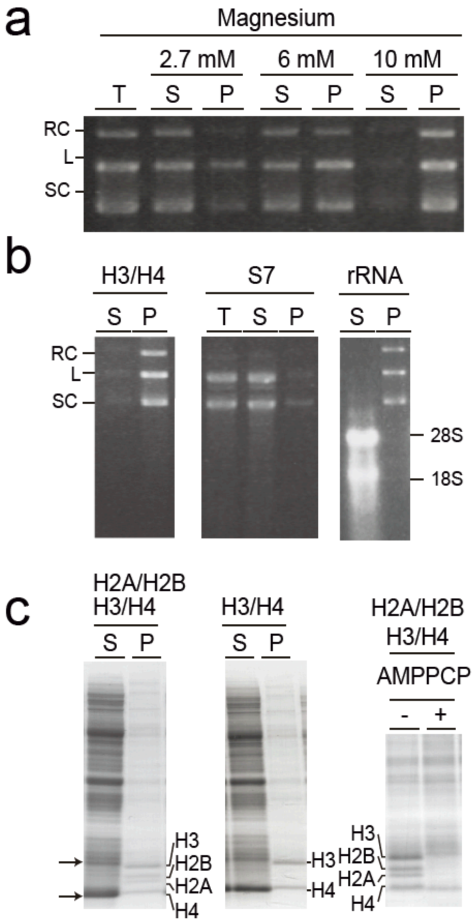
a
Magnesium
2.7 mM
6 mM
10 mM
T
S
P
S
P
S
P
RC
L
SC
b
H3/H4
S7
rRNA
S
P
T
S
P
S
P
RC
L
SC
28S
18S
c
H2A/H2B
H3/H4
S
P
H3/H4
S
P
H2A/H2B
H3/H4
AMPPCP
-
+
H3
H2B
H2A
H4
H3
H4
H3
H2B
H2A
H4

**Figure S6**

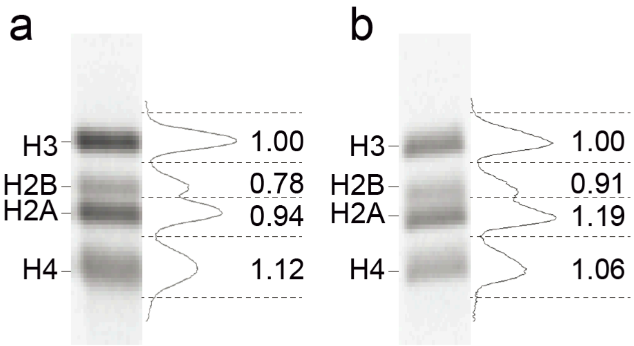

**Figure S7**

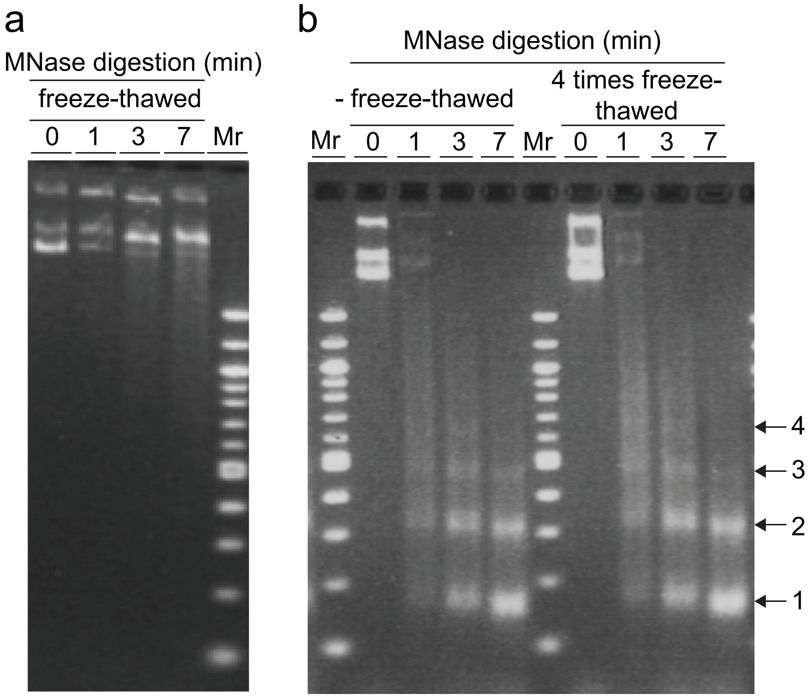
a
MNase digestion (min)
freeze-thawed
0 1 3 7 Mr
b
MNase digestion (min)
- freeze-thawed
4 times freeze-thawed
Mr 0 1 3 7 Mr 0 1 3 7
4
3
2
1

**Figure S8**

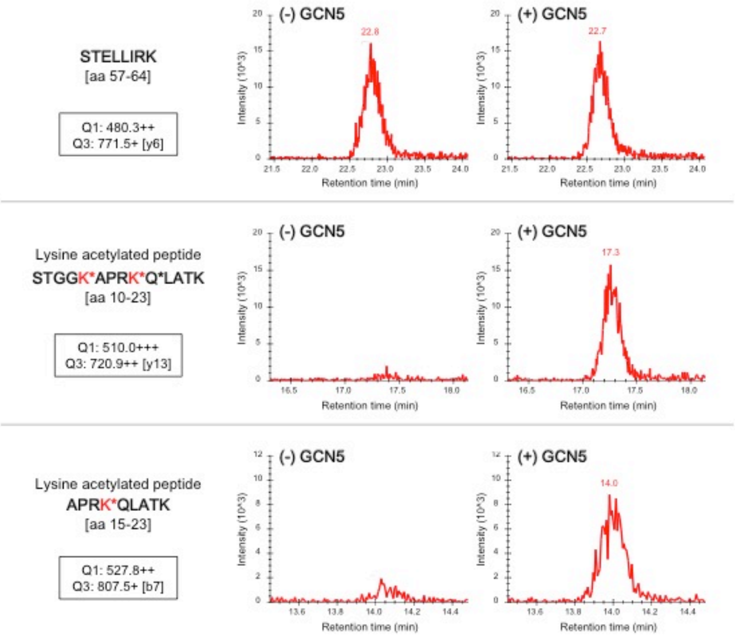

STELLIRK
[aa 57-64]
Q1: 480.3++
Q3: 771.5+ [y6]
Lysine acetylated peptide
STGGK*APRK*Q*LATK
[aa 10-23]
Q1: 510.0+++
Q3: 720.9++ [y13]
Lysine acetylated peptide
APRK*QLATK
[aa 15-23]
Q1: 527.8++
Q3: 807.5+ [b7]
(-) GCN5
(+) GCN5
22.8
22.7
17.3
14.0
Intensity (10^3)
Retention time (min)

# Figure S9

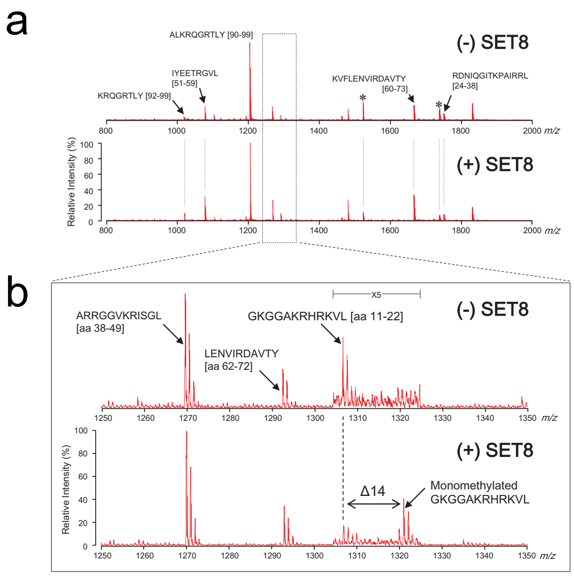
a
ALKRQGRTLY [90-99]
(-) SET8
IYEETRGVL
[51-59]
KRQGRTLY [92-99]
KVFLENVIRDAVTY
[60-73]
RDNIQGITKPAIRRL
[24-38]
(+) SET8
Relative Intensity (%)
m/z
b
x5
ARRGGVKRISGL
[aa 38-49]
GKGGAKRHRKVL [aa 11-22]
LENVIRDAVTY
[aa 62-72]
Δ14
Monomethylated
GKGGAKRHRKVL
